# Psychobiotic, nutritional, and behavioral interventions targeting the microbiome-gut-brain axis in depression, anxiety, and stress: a scoping review

**DOI:** 10.3389/fnut.2026.1856471

**Published:** 2026-09-04

**Authors:** Qiuhua Dai, Chao Fang, Yajun Du, Haoran Sun, Zhongyu Duan

**Affiliations:** 1 Yunnan Key Laboratory of Dai and Yi Medicines, Yunnan University of Chinese Medicine, Kunming, China; 2 Haikou Hospital of Traditional Chinese Medicine, Haikou, China

**Keywords:** anxiety, depression, dietary intervention, microbiome-gut-brain axis, probiotics, psychobiotics, scoping review, stress

## Abstract

Probiotics and psychobiotics are increasingly investigated as microbiome-gut-brain axis (MGBA)-targeted strategies for managing depression, anxiety, and stress-related symptoms, but human evidence remains fragmented across intervention types and biomarker domains. We conducted a scoping review of human intervention studies to map MGBA-targeted psychobiotic, nutritional, dietary, and behavioral interventions, with a specific focus on symptom-biomarker bridging evidence. Searches of major biomedical and multidisciplinary databases combined key terms for the microbiome/gut-brain axis, depression/anxiety/stress, probiotics/psychobiotics, dietary or behavioral interventions, and biomarker domains. The search yielded 1,390 records; 72 full-text reports were assessed; 32 original reports were retained after report-level adjudication; and, after study-family consolidation, 30 independent human studies were included in the study-level synthesis. Studies were classified as direct bridge, parallel evidence, or no bridge according to whether biomarker changes were statistically linked to emotional outcomes. Most included studies focused on probiotic or psychobiotic interventions, with fewer studies examining prebiotics, synbiotics, short-chain fatty acid approaches, dietary interventions, or mind-body/behavioral strategies. Across the 30 studies, 9 were classified as direct bridge, 18 as parallel evidence, and 3 as no bridge. The most frequently examined biomarker domains were gut microbiota, microbial metabolites, and inflammation/immune markers, with common specific readouts including BDNF, 5-HT/serotonin-related markers, IL-6, TNF-α/CRP, cortisol/CAR, and SCFA/metabolite measures. Overall, MGBA-related nutritional and psychobiotic interventions may contribute to mood-symptom management by modulating microbial, metabolic, immune, neuroendocrine, and neurotrophic pathways, but robust symptom-biomarker bridging evidence remains limited. Future trials should predefine bridge hypotheses, standardize core biomarker domains and sampling time points, and clearly distinguish primary trial reports from linked secondary publications.

## 1 Introduction

Mood and behavior are increasingly studied through the microbiome-gut-brain axis (MGBA), a framework linking diet, gut microbial ecology, host metabolism, immune activity, autonomic signaling, and neural function. Within this model, gut microbiota and their metabolites may influence depression, anxiety, and stress-related responses through interacting inflammatory-immune, neuroendocrine, autonomic, neurotransmitter, and neurotrophic pathways (1–3). This framework has shaped research in nutrition, probiotics, functional foods, and mind-body approaches. A growing number of human intervention studies now test whether targeted approaches can regulate MGBA-related pathways and improve emotional outcomes. These approaches include probiotics, psychobiotics, prebiotics, synbiotics, short-chain fatty acid strategies, dietary patterns, and behavioral or mind-body strategies; in the included synthesis, the latter were represented mainly by mindfulness-based cognitive therapy and mind-regulating acupuncture (3, 4).

The term “psychobiotics” initially referred to microbial interventions designed to improve mental states through actions on the microbiota-gut-brain axis. Functionally, candidate psychobiotics may support therapeutic potential in mental health disorders by reshaping microbial composition, increasing beneficial microbial metabolites and neuroactive substances, influencing tryptophan-serotonin and gamma-aminobutyric acid-related signaling, strengthening barrier-related functions, and modulating immune, cytokine, inflammatory, and stress-endocrine pathways (1–3). A recent systematic literature review further summarized promising effects of probiotics/psychobiotics on stress, mood, and depression, while emphasizing limitations related to clinical outcomes, sample size, and variable dosing (5). Human studies have also expanded to include standard probiotics originally developed for gut health but now evaluated for emotional outcomes. For evidence mapping, this review therefore groups probiotics and psychobiotics into a single “probiotic/psychobiotic” category while retaining the original study context in interpretation.

A central challenge in human MGBA intervention research is determining whether biological changes are linked to emotional improvement rather than merely measured alongside symptom scales. Many studies report mood outcomes and biomarkers in the same trial, but only a subset formally tests whether changes in microbiota, metabolites, inflammatory markers, neuroendocrine markers, neurotransmitter/trophic markers, or neural readouts are associated with symptom changes. A single intervention may also generate *post-hoc* analyses, preliminary biomarker reports, mechanistic follow-up reports, or companion papers. Without study-family adjudication, the number of publications may be mistaken for the number of independent studies.

The objective of this scoping review was to map human MGBA-targeted intervention studies at the study level, identify which intervention categories were linked to which objective biomarker domains and specific biomarkers, evaluate the maturity of symptom-biomarker bridging evidence, and distinguish primary trial reports from linked secondary publications within the same study family.

## 2 Materials and methods

### 2.1 Review design and research question

This study was designed as a scoping review of human intervention studies and is reported in accordance with PRISMA-ScR, with selected PRISMA 2020 workflow elements additionally applied to enhance screening transparency (6–8). The review question was defined using a PICOS-informed framework. Population: human participants with depression, anxiety, stress-related symptoms, subclinical emotional symptoms, or clinically relevant high-stress/medical populations reporting emotional outcomes. Interventions: microbiome-gut-brain axis-oriented nutritional, psychobiotic, probiotic, prebiotic, synbiotic, dietary, microbial, or behavioral interventions. Comparators: placebo, usual care, active control, wait-list, or study-specific within-group pre-post comparisons, depending on design. Outcomes: depression-, anxiety-, stress-, or related emotional outcomes together with at least one objectively measured biomarker available within the same study family. Study designs: original human interventional studies. Owing to substantial heterogeneity in populations, interventions, biomarker domains, follow-up duration, outcome measures, and analytical approaches, no pooled meta-analysis was performed; instead, the review adopted a study-level evidence-mapping approach focused on the maturity of symptom-biomarker bridging evidence.

### 2.2 Inclusion and exclusion criteria

Inclusion criteria: ① Original human intervention studies; ② Interventions targeting MGBA-related nutritional, psychological, probiotic, dietary, microbial, or behavioral pathways; ③ Reporting at least one outcome on depression, anxiety, stress, or similar emotional/stress-related outcomes; ④ Studies where objective biomarker data can be extracted within the same study family. For *post-hoc*, preliminary, subreports, or mechanistic follow-up reports from the same study family, these were placed in the supplementary materials audit layer only if they avoided duplicate counting and provided essential mechanistic information.

Exclusion criteria: animal studies, observational studies, reviews, meta-analyses, conference abstracts, or registrations, and full texts lacking target emotional outcomes or extractable biomarker data.

### 2.3 Information sources and search strategy

The literature search was conducted across five electronic databases: PubMed, Embase, Web of Science, Cochrane CENTRAL, and Scopus. The search included studies published between January 2014 and April 2026, with the final search completed on 7 April 2026. To find relevant human studies, the search strategy combined terms on the microbiome-gut-brain axis, depression, anxiety, stress-related outcomes, intervention types, and biomarker domains. The purpose was to identify studies that could provide evidence on symptom-biomarker bridging. The full search strategies for all databases are shown in Supplementary Table S1.

### 2.4 Study selection, screening workflow, and study-family adjudication

All retrieved records were imported into EndNote, and duplicates were removed. Titles and abstracts of the remaining records were screened, followed by full-text review and verification of main extraction items. Two reviewers independently completed each step using standardized forms. If they disagreed, they discussed the case; unresolved disagreements were decided by a third reviewer. To prevent double-counting, the review did not treat every publication as a separate study. Each report’s eligibility was assessed independently, and those from the same trial were grouped into a single study family for the main synthesis. This was important because a single trial could yield several related papers. Some were early biomarker papers, others were later mechanistic analyses or companion publications. When such reports offered background or extra detail but failed to qualify as independent studies, they were kept only in the supplementary audit layer and not included in the main denominator. The final substantive synthesis was therefore conducted at the study level rather than the publication level.

### 2.5 Data extraction, bridging definitions, and study-level processing principles

A standardized extraction form was used to record authors, year, country, population, sample size, design, intervention, comparison method, duration, emotional outcomes, biomarker domains, specific biomarker readouts, bridge classification, and key study-level notes. Manuscript-level harmonization was performed for biomarker domains, which were uniformly consolidated into 7 high-level domains: gut microbiota, microbial metabolites, inflammation/immune, neuroendocrine/autonomic, neurotransmitters/trophic, neuroimaging, and barrier/permeability. Specific biomarkers recorded in Supplementary Table S2 were retained to support the revised biomarker-level evidence matrix.

Bridge maturity was classified into three categories. Direct bridge indicated that an explicit statistical link between biomarker change and symptom/emotional change was tested within the same study-level record, for example through correlation, regression, responder/stratification analysis, moderation analysis, or another formal association model. Parallel evidence indicated that both psychological outcomes and objective biomarkers were collected and reported within the same study family, but no formal symptom-biomarker linkage test was performed. No bridge did not mean that emotional outcomes and biological measures were necessarily absent or collected in completely separate contexts; rather, it indicated that no interpretable symptom-biomarker association could be established, such as when only one side of the symptom/biomarker signal changed, timing was not comparable, or the study did not provide analyzable evidence linking the two. Design-sensitive direct signals (e.g., responder-based MBCT or neurophysiological vitality coupling) were further labeled as “design-sensitive direct bridge” in the Supplementary material.

### 2.6 Principles for addressing risk of bias and uncertainty in the evidence

Given the high heterogeneity in study designs among the included studies in this review (including randomized controlled trials, single-arm trials, open-label trials, add-on trials, and post-hoc/mechanism follow-up analyses), this study did not perform a mechanical meta-analysis using a single quantitative scoring tool for all studies. Instead, we adopted a structured narrative bias audit strategy tailored to the specific study design. For randomized controlled trials and non-randomized studies, we provided qualitative descriptions based on the core dimensions of RoB 2 (9) and ROBINS-I (10), respectively; for designs such as open-label, single-arm, and proof-of-concept studies, we supplemented these with narrative bias notes specific to their design. Risk of bias, design limitations, linked secondary reports, indirectness, and imprecision in sample size were collectively used to explain study-level evidence uncertainty. Itemized audit results are presented in Supplementary Table S5.

## 3 Results

### 3.1 Screening process and study-level consolidation results

**Figure 1:**
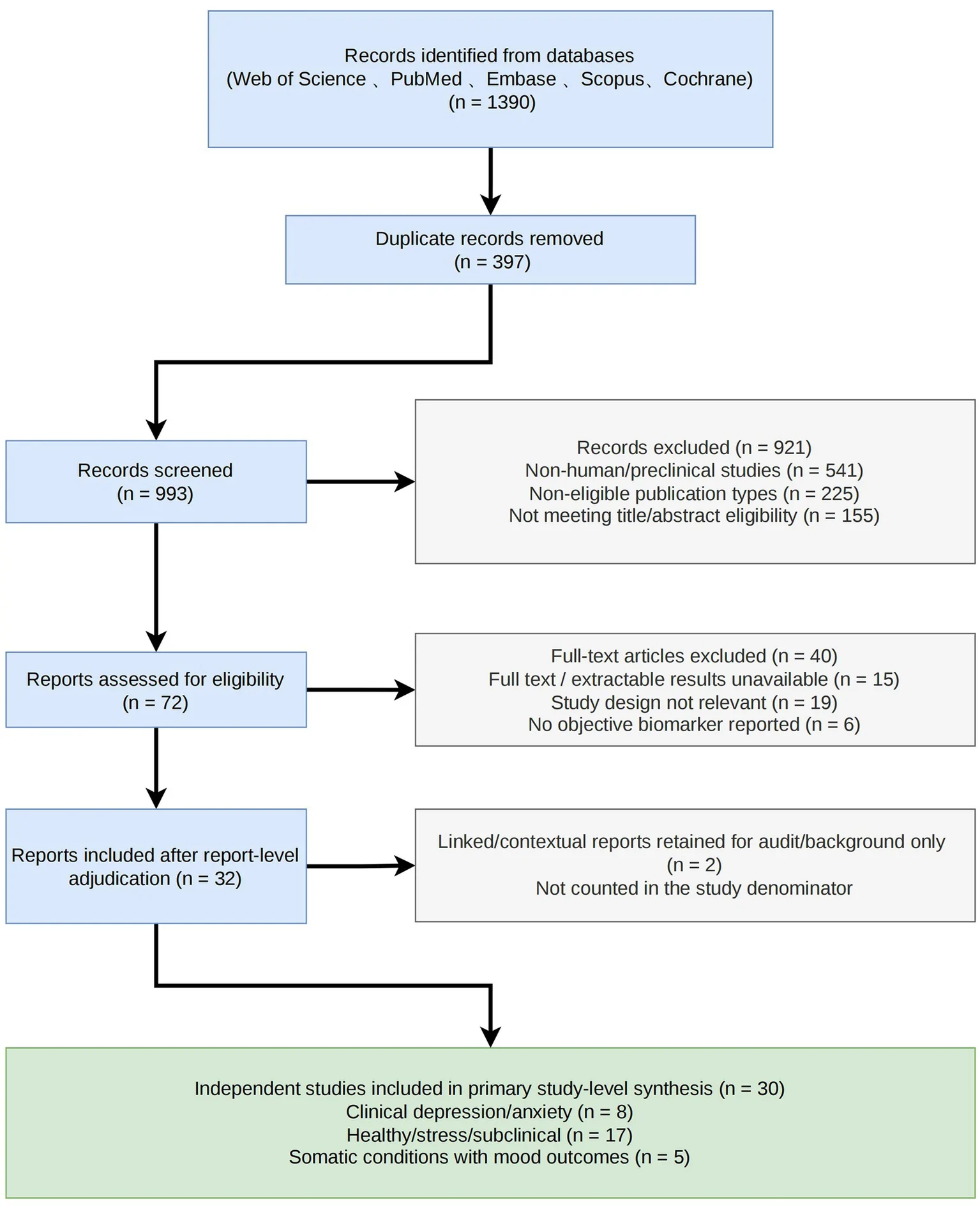
PRISMA-style flow diagram summarizing record identification, screening, full-text eligibility assessment, report-level adjudication, and final study-family consolidation. Linked/companion reports retained only for audit or context were not counted in the denominator of 30 independent studies.

A total of 1,390 records were retrieved; after deduplication, 993 records proceeded to title and abstract screening; 72 full-text reports were included in the assessment; among these, 32 original reports passed report-level eligibility adjudication, while the remaining 40 full-text reports were excluded from the main analysis. To avoid double-counting, following further study-family consolidation, 2 reports (1 preliminary biomarker sub-report and 1 accompanying mechanism report) were listed separately as background review materials. Ultimately, exactly 30 independent human studies were included in the substantive main study-level synthesis. The specific screening process is shown in Figure 1.

### 3.2 Overall characteristics of included studies (study-level)

The final 30 included studies were published between 2019 and 2025. At the study-level, 17 studies involved healthy/high-stress/subclinical samples, 8 studies involved clinical depression or anxiety samples, and 5 studies involved samples with comorbid physical diseases but reported emotional outcomes. The primary intervention type was probiotic/psychobiotic (20/30), followed by prebiotic/synbiotic/SCFA (4/30), mind-body/behavioral (2/30), dietary/food-based (2/30), and mixed multi-component (2/30).

Table 1 provides an intervention-category overview of the included studies, including the first author and publication year of each study, the number of independent studies, bridge maturity, summary population/design features, and common biomarker readouts. Full study-level characteristics, specific biomarkers, and study-family information are provided in Supplementary Tables S2–S4. The 32 source reports, including the 30 independent studies and 2 linked/contextual reports, are listed in Supplementary Table S3 (11–42). This organization avoids a lengthy narrative author-by-author listing while preserving study-level traceability in the main text.

**Table 1:** Overview of the 30 independent human intervention studies by intervention category.

Intervention category	Study	Studies, *n* (%)	Bridge maturity	Summary of population/design features and common biomarker readouts
Probiotic/psychobiotic	Rudzki (18); Wang (30); Gualtieri (16); Heidarzadeh-Rad (15); Liu (25); Reininghaus (17); Chen (19); Kim (24); Ma (26); Nobile (11); Rahimlou (12); Rode (21); Schaub (13); Sun (29); Obermoser (27); Tavares-Silva (28); Ahmad (14); Guan (23); Rode (22); Tie (20)	20 (66.7)	7 direct; 12 parallel; 1 no bridge	Largest evidence cluster, including clinical depression/anxiety, high-stress/subclinical, older-adult, and somatic-disease samples; mainly randomized or add-on trials. Common readouts included microbiota/sequencing, metabolites/SCFAs, inflammatory markers (IL-6, TNF-α, CRP), BDNF, 5-HT/serotonin-related markers, and neuroimaging.
Prebiotic/synbiotic/SCFA	Mysonhimer (31); Dalile (32); Jackson (33); Hall (34)	4 (13.3)	1 direct; 1 parallel; 2 no bridge	Mostly healthy/stress or metabolic-risk samples using crossover or pilot randomized designs. Biomarkers included microbiota profiling, SCFAs/metabolites, cortisol/CAR, BDNF, and hs-CRP.
Mind-body/behavioral	Wang (36); Xie (35)	2 (6.7)	1 direct; 1 parallel; 0 no bridge	Included mindfulness-based cognitive therapy and mind-regulating acupuncture. Readouts included fecal microbiota, tryptophan-pathway prediction, serum 5-HT, and BDNF.
Dietary/food-based	Herselman (37); Berding (38)	2 (6.7)	0 direct; 2 parallel; 0 no bridge	Included walnut supplementation and a psychobiotic-diet intervention. Biomarkers included gut microbial diversity/composition, metabolomics or metabolic markers, cortisol, and other stress biomarkers.
Mixed multi-component	Talbott (39); Talbott (40)	2 (6.7)	0 direct; 2 parallel; 0 no bridge	Coordinated pro-/pre-/phyto-biotic regimens. Readouts included PCR-based microbiome balance, butyrate kinase, cortisol, and glucose/lipid-related markers.

Studies are summarized at the intervention-category level. Included studies are identified by first author and publication year; full bibliographic details are provided in the reference list. Biomarker readouts were harmonized from Supplementary Table S2. A single study may contribute to more than one biomarker category. Direct, parallel, and no-bridge classifications are defined in Section 2.5 and detailed in Supplementary Table S4.

Therefore, the following structured frequencies should be interpreted as study-level counts rather than article counts. See Table 1 and Supplementary Tables S2–S4 for details.

### 3.3 Distribution of biomarker domains and locations with the highest density of linkages

After harmonization, the three most evidence-rich biomarker domains were gut microbiota (19 studies), microbial metabolites (14 studies), and inflammation/immune markers (13 studies), followed by neurotransmitters/trophic markers (11 studies), neuroendocrine/autonomic markers (8 studies), neuroimaging markers (4 studies), and barrier/permeability markers (1 study). This pattern indicates that current human studies have generally incorporated microbiota, metabolites, and inflammation/immunity into the evaluation of emotion-related interventions, but indicators related to the intestinal barrier remain notably scarce (Table 2).

**Table 2:** Distribution of bridge evidence categories by intervention type (study-level).

Intervention type	Direct bridge	Parallel evidence	No bridge	Total
Probiotic/psychobiotic	7	12	1	20
Prebiotic/synbiotic/SCFA	1	1	2	4
Mind-body/behavioral	1	1	0	2
Dietary/food-based	0	2	0	2
Mixed multi-component	0	2	0	2
Total	9	18	3	30

“Direct bridge” refers to studies that have explicitly statistically linked “biomarker changes—symptom changes” within the same study-level record, such as through correlation, regression, responder stratification, or moderation analyses; “Parallel evidence” refers to studies in which psychological outcomes and objective biomarkers were collected and reported within the same study family, but no formal symptom-biomarker linkage test was performed; “No bridge” refers to cases where no interpretable link has been established or only a one-sided signal is observed.

**Figure 2:**
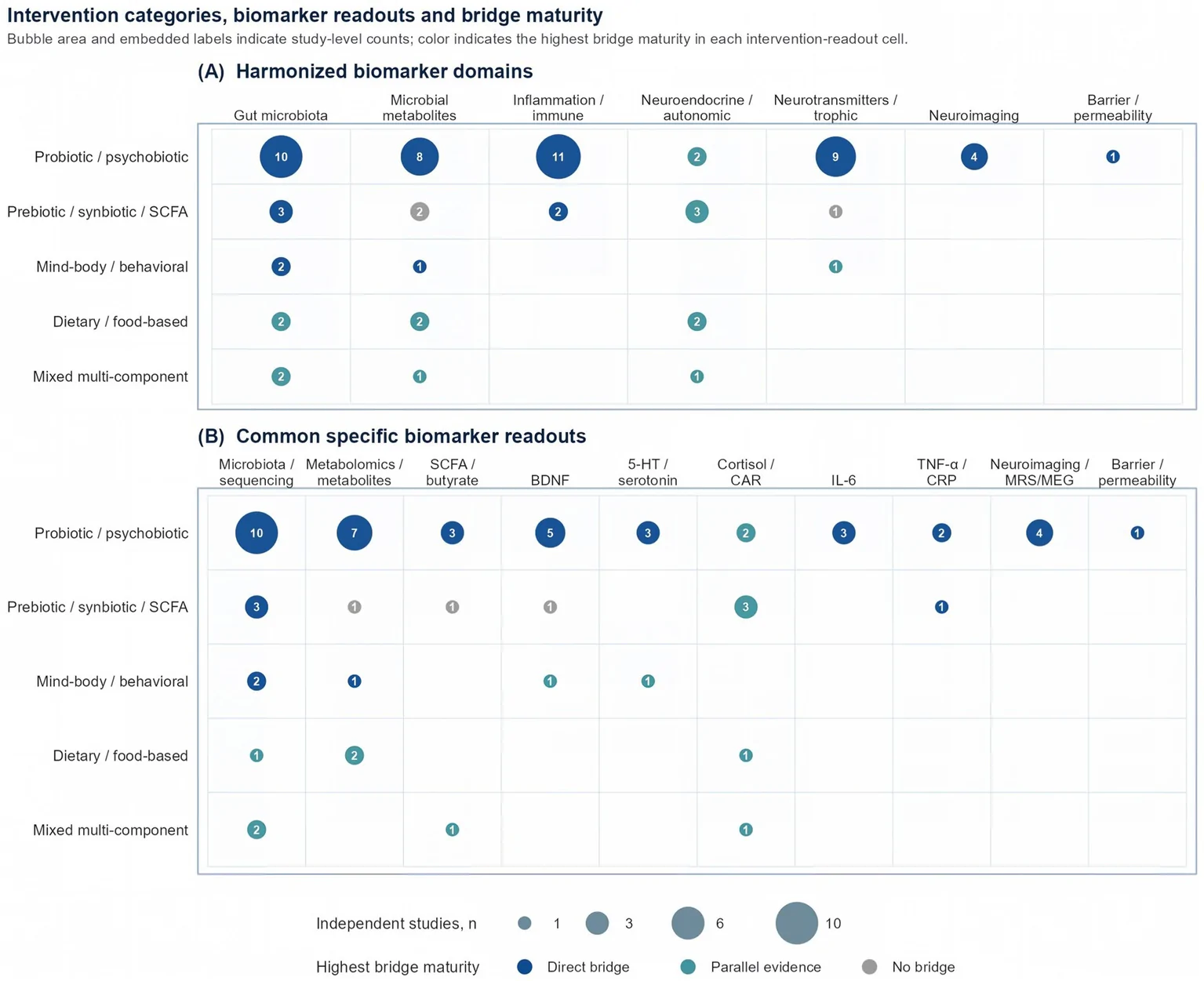
Two-panel study-level evidence matrix of intervention categories, biomarker readouts, and bridge maturity. Panel **(A)** shows harmonized biomarker domains; Panel **(B)** shows common specific biomarker readouts derived from Supplementary Table S2, including BDNF, 5-HT/serotonin-related markers, IL-6, TNF-α/CRP, cortisol/CAR, SCFA/metabolite measures, neuroimaging, and barrier/permeability markers. Bubble size and embedded labels represent the number of independent studies in each cell; color represents the highest bridge maturity observed in that cell (direct pbridge > Parallel nevidence > No bridge).

In terms of intervention types, probiotic/psychobiotic studies not only accounted for the largest number but also covered the broadest range of biomarker domains; prebiotic/synbiotic/SCFA studies primarily focused on microbiota, metabolites, and stress-endocrine pathways; mind-body/behavioral studies were fewer in number, but the MBCT responder analysis within this category suggests that this pathway warrants further exploration in bridge research (36). The study-level cross-distribution of intervention categories, harmonized biomarker domains, common specific biomarkers, and bridge maturity is shown in Figure 2.

At the specific biomarker level, microbiota or sequencing-based readouts were the most frequent specific readout group, followed by metabolomics/metabolite measures. Among common marker classes, BDNF was reported in 7 studies, 5-HT/serotonin-related markers in 4 studies, IL-6 in 3 studies, and TNF-α/CRP-related inflammatory markers in 3 studies. Cortisol/CAR readouts were also common (7 studies), while barrier/permeability markers were rare and appeared in only 1 study. These marker-level distributions indicate that the literature is concentrated around microbial composition, microbial metabolites, inflammatory markers, neurotrophic markers, serotonin-related signaling, and stress-endocrine readouts, but that most marker classes remain reported as parallel evidence rather than formal bridging evidence.

### 3.4 Distribution of bridge maturity (study-level)

Classified by study level, among the 30 included studies, 9 had a Direct bridge, 18 had Parallel evidence, and 3 had no bridge. Overall, MGBA intervention studies have generally entered the stage of jointly measuring “emotional outcomes + objective biomarkers,” but studies that have truly established an explicit symptoms-biomarker bridge remain in the minority. It should be emphasized that the count here is based on independent studies; linked secondary reports are not counted twice.

### 3.5 Linked reports and design-sensitive bridge evidence

A full-text review of each report revealed that not all source reports were standard parallel-group confirmatory RCTs. The most clearly defined linked-study family appeared in PROVIT: Reininghaus 2020 served as the main trial report, while Reiter 2020 was a preliminary subreport on IL-6 biomarkers from the same study (17, 41). In this study-level consolidation, the two were merged into a single independent study and are no longer double-counted.

Additionally, Liu 2020 and Ma 2021 both fall under the category of follow-up/secondary mechanistic reporting: the former is a mechanistic extension of the DR7 trial, while the latter is a metagenomic follow-up of the P-8 trial (25, 26). Heidarzadeh-Rad 2020, on the other hand, is a clear *post-hoc* biomarker analysis (15). These reports can still contribute bridging or mechanistic information, but in terms of evidence interpretation, they are all treated as “design-sensitive study-level proxies.”

To reduce over-interpretation, this study has applied additional labeling to the most marginal entries in the “direct bridge” category. The direct signal in Wang 2022 primarily stems from MBCT responder analysis and microbiota responsiveness, constituting a design-sensitive direct bridge (36); the direct signal in Wang 2019 is mainly manifested as the association between neurophysiological changes and changes in vitality/fatigue, also representing a neurophysiological direct bridge rather than a typical symptom-mediated model (30).

Raman 2023 was not included in the denominator of the main studies, but was retained in the supplementary materials as a contextual, mechanistic companion report to illustrate meditation-related microbiome/metabolome clues (42). This approach prevented linked reports from inflating the number of independent studies in the main synthesis.

### 3.6 Narrative assessment of risk of bias and uncertainty in the evidence

The included studies were mainly randomized controlled trials (RCTs), although some were open-label, single-arm, adjunctive, *post-hoc*, or mechanistic follow-up studies. Key uncertainties arise from small sample sizes, short follow-up periods, inconsistent comparison methods, unregistered bridge hypotheses, and study-level proxies derived from linked secondary reports. Overall confidence in the current evidence, particularly in the proposed causal bridging chain, is therefore limited. These statements represent narrative uncertainty judgments rather than a formal GRADE assessment. See Supplementary Table S5 for a detailed audit.

## 4 Discussion

### 4.1 Principal findings

Three main findings emerged from this review. First, human studies in this field have moved beyond symptom reporting alone and increasingly assess emotional outcomes together with microbiota-, metabolite-, immune-, neuroendocrine-, neurotrophic-, or brain-related biomarkers. Second, the most concentrated human evidence concerns probiotic and psychobiotic interventions, which accounted for two-thirds of the included studies and covered the broadest range of biomarker domains (11–30).

Third, the literature remains more mature in collecting biomarkers than in demonstrating symptom-biomarker bridging: most studies reported parallel evidence, whereas formal links between biological change and improvement in depression, anxiety, stress, or related mood outcomes were less common (13, 15, 16, 30, 34, 36). These findings place the field between biological plausibility and clinically actionable mechanism: MGBA-targeted interventions appear promising, especially probiotic/psychobiotic approaches, but the mechanistic chain from intervention to biomarker change to emotional benefit is still incompletely established.

### 4.2 Interpretation of the current evidence

**Figure 3:**
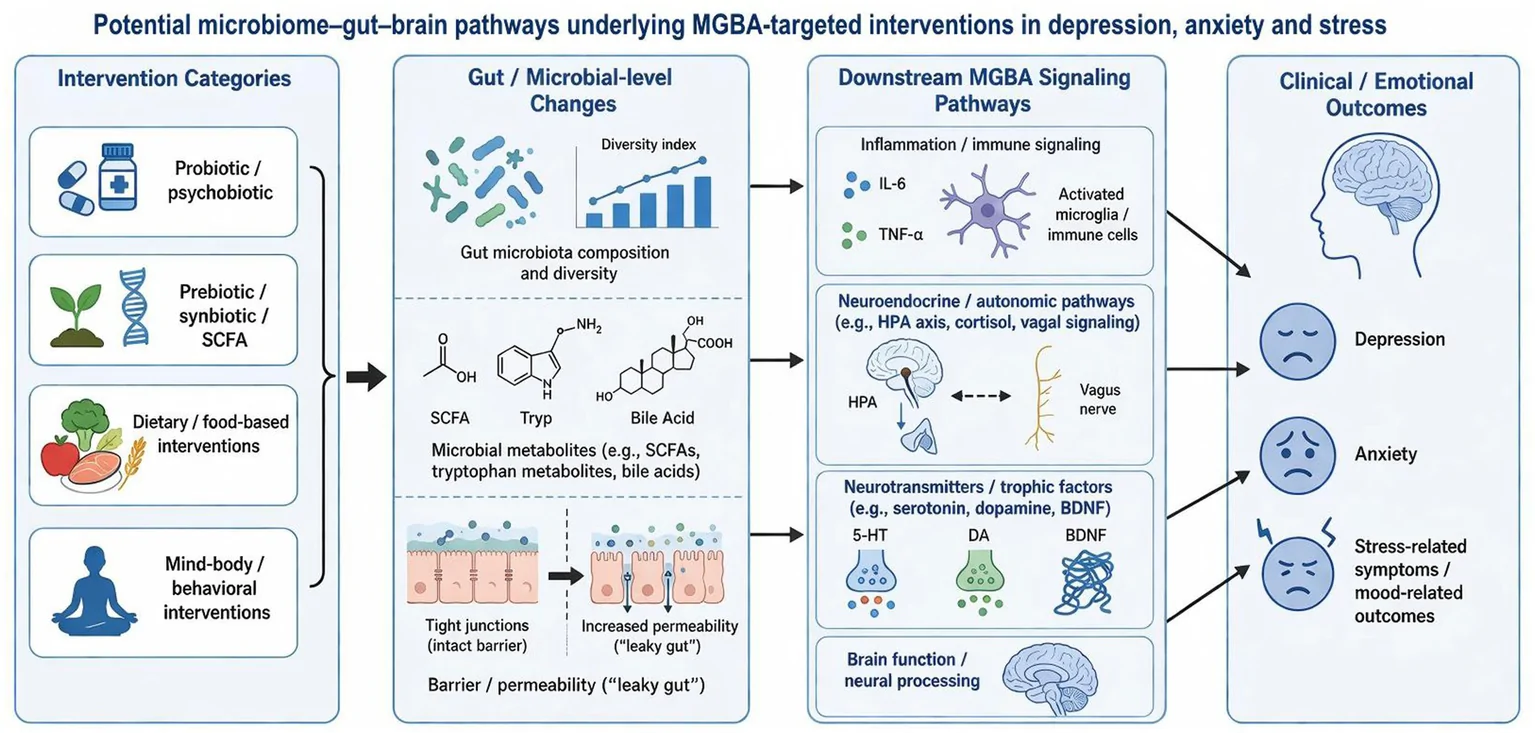
Conceptual schematic of plausible microbiome-gut-brain axis pathways relevant to the included intervention literature. The figure summarizes intervention categories, potential gut/microbial-level changes, downstream signaling pathways, and mood-related outcomes discussed across the reviewed human studies. It is intended as an interpretive framework rather than a quantitative evidence map or a direct summary of causal proof.

From a nutrition and brain-health perspective, the current evidence supports a cautious interpretation that probiotics, psychobiotics, and related nutritional interventions may contribute to the management of stress-, anxiety-, and depression-related outcomes through MGBA-linked pathways. Across the included probiotic/psychobiotic trials, symptom-related benefits were most often interpreted alongside changes in gut microbial composition, microbial metabolites, inflammatory markers such as IL-6, TNF-α, or CRP, neurotrophic markers such as BDNF, serotonin/5-HT-related measures, cortisol/CAR, or neuroimaging readouts. This pattern is consistent with current MGBA models in which microbial, metabolic, immune, neuroendocrine, autonomic, and neurotransmitter/trophic pathways interact rather than operate as isolated mechanisms (1–4). However, the evidence should not be read as proof that any single biomarker explains clinical benefit across populations. Figure 3 is therefore retained as a conceptual schematic of plausible pathways, whereas the revised Figure 2 provides the data-derived evidence map across intervention categories, biomarker domains, specific biomarkers, and bridge maturity.

### 4.3 Limitations of the current evidence base

The current evidence base has several limitations that restrict clinical interpretation. Study populations were highly heterogeneous, including patients with major depressive disorder or anxiety, healthy individuals under stress, older adults, patients with post-stroke depression, people with coronary artery disease, and people with post-infectious fatigue. Study designs also varied, including randomized controlled trials, adjunctive trials, open-label trials, single-arm studies, *post hoc* biomarker analyses, and linked mechanistic follow-up reports. This heterogeneity limits direct comparison of effect sizes and makes it difficult to infer a unified clinical treatment pathway.

Among the nine studies marked as “Direct Bridge,” the strength and type of statistical evidence varied widely. Most bridge evidence came from change-score correlations, exploratory regression or multivariable association analyses, responder-based analyses, or design-sensitive neurophysiological associations. Formal mediation analyses that could test whether biomarker change statistically accounted for symptom improvement were generally absent. Therefore, direct bridge should be interpreted as evidence of an explicit symptom-biomarker association within a study, not as definitive causal mediation.

Evidence quality was also uneven across intervention types. Probiotic interventions are comparatively easier to test in double-blind, placebo-controlled designs, whereas dietary and behavioral interventions are more difficult to blind, standardize, and isolate mechanistically. Within probiotic/psychobiotic studies, clinical translation is further constrained by variability in strain composition, dose, dosing frequency, treatment duration, adherence assessment, comparator choice, baseline diet control, and biomarker sampling time points. These differences make it difficult to determine whether inconsistent results reflect biological heterogeneity, intervention design, insufficient duration, underpowered biomarker analyses, or lack of prespecified bridge hypotheses.

### 4.4 Implications for future trial design

Future trials should prioritize prespecified symptom-biomarker hypotheses over broad exploratory biomarker panels, with biological sampling aligned to emotional outcome assessment. Future studies should prespecify a primary emotional outcome, a primary biomarker domain or marker, the expected direction of change, and the statistical model used to test bridging.

For probiotic and psychobiotic trials, reporting should include strain identity, colony-forming unit dose or equivalent viable-dose information, dosing frequency, formulation, duration, adherence, diet or medication restrictions, and sampling time points. For dietary and behavioral interventions, protocols should define the active components, delivery frequency, exposure intensity, adherence metrics, and contextual co-interventions. Across all intervention categories, biomarker sampling should be synchronized with symptom assessment so that parallel evidence can be upgraded to direct bridge testing when appropriate.

Reporting also needs to distinguish primary trial reports from secondary analyses, mechanistic follow-up reports, and *post-hoc* publications. This distinction is essential for scoping reviews because linked reports can add mechanistic detail but should not inflate the denominator of independent studies. The supplementary tables Therefore organize study-level characteristics, study-family relationships, bridge adjudication, and uncertainty notes in a harmonized chronological order.

A persistent mechanistic gap concerns intestinal barrier status. Although the “leaky gut” hypothesis is frequently proposed as a link between gut dysbiosis, systemic inflammation, and mood-related symptoms, only one included study directly assessed barrier or permeability-related markers. This gap weakens the ability to connect microbial change to downstream immune and neuroendocrine pathways in a complete causal sequence.

The shortage of barrier evidence is partly methodological. Gold-standard permeability approaches, such as disaccharide absorption testing, require burdensome sample collection, whereas blood-based approaches such as serum zonulin are easier to implement but remain debated in specificity. Future MGBA trials should therefore prioritize feasible, validated, and low-burden barrier/permeability measures when this pathway is central to the proposed mechanism. Such methodological standardization would help move the field from parallel biological plausibility toward reproducible symptom-biomarker bridging.

## 5 Conclusion

In summary, human studies on MGBA-targeted interventions support an emerging nutritional psychiatry framework in which emotional outcomes are increasingly evaluated together with biological readouts. After adjudication and study-family consolidation, 30 independent human studies formed the main synthesis, with the strongest evidence concentrated in probiotic/psychobiotic interventions and in gut microbiota, microbial metabolite, and inflammation/immune domains. Specific biomarker readouts such as BDNF, 5-HT/serotonin-related markers, IL-6, TNF-α/CRP, cortisol/CAR, and SCFA/metabolite measures were recurrent but were often reported without formal symptom-biomarker linkage. Current evidence supports biological plausibility, but it does not yet establish a reproducible biomarker-mediated pathway from intervention exposure to emotional improvement. Future trials should predefine bridge hypotheses, standardize intervention and biomarker reporting, and align biological sampling with symptom assessment.

## Data Availability

The original contributions presented in the study are included in the article/Supplementary material, further inquiries can be directed to the corresponding author.
